# Autonomous Manoeuvring Systems for Collision Avoidance on Single Carriageway Roads

**DOI:** 10.3390/s121216498

**Published:** 2012-11-29

**Authors:** Felipe Jiménez, José Eugenio Naranjo, Óscar Gómez

**Affiliations:** University Institute for Automobile Research (INSIA), Technical University of Madrid (UPM), 28040 Madrid, Spain; E-Mails: joseeugenio.naranjo@upm.es (J.E.N.); labie.insia@upm.es (O.G.)

**Keywords:** intelligent transport systems, laser scanner, cooperative warning, wireless communications

## Abstract

The accurate perception of the surroundings of a vehicle has been the subject of study of numerous automotive researchers for many years. Although several projects in this area have been successfully completed, very few prototypes have actually been industrialized and installed in mass produced cars. This indicates that these research efforts must continue in order to improve the present systems. Moreover, the trend to include communication systems in vehicles extends the potential of these perception systems transmitting their information via wireless to other vehicles that may be affected by the surveyed environment. In this paper we present a forward collision warning system based on a laser scanner that is able to detect several potential danger situations. Decision algorithms try to determine the most convenient manoeuvre when evaluating the obstacles’ positions and speeds, road geometry, *etc.* Once detected, the presented system can act on the actuators of the ego-vehicle as well as transmit this information to other vehicles circulating in the same area using vehicle-to-vehicle communications. The system has been tested for overtaking manoeuvres under different scenarios and the correct actions have been performed.

## 1. Introduction

Recent decades have seen an enormous growth in mobility, a large part of which has been absorbed by road transport [1]. This situation has given rise to various negative effects, such as accidents, congestion and contamination, *etc.* Throughout the years numerous measures have been applied to improve road transport. However, in some contexts, it has now become practically impossible to improve on the classic solutions and any progress may seem little. This has led to the introduction of alternatives under the name of Intelligent Transport Systems (ITS), which is the name given to applications that integrate communications and information control and processing into transport systems. Their fundamental objectives, among others, are to reduce accidents, save energy, reduce pollution and increase the efficiency of the transport system [2].

The classic way of classifying safety systems makes a distinction between active safety systems (aimed at reducing the number of accidents) and passive safety (aimed at reducing the consequences of accidents). However, more complex models denominate these groups of systems as primary and secondary systems, respectively, adding other groups, such as driver assistance and tertiary safety, which give rise to pre-collision systems [3,4]. These systems use information captured by the sensors so that they can act on the control and protection systems in order to reduce the probability and consequences of the accident. They allow taking measures some seconds in advance, allowing new measures to be taken and/or increasing their effectiveness. Some of the actions are automatic braking, automatic action on the steering system to improve the angle of impact, pretensioner activation, preparation of airbags and measures to improve the compatibility between vehicles, such as extendable bumpers, suspension height control, *etc.*, or the deployment of measures to minimize the effects of pedestrian accidents. There are two critical aspects in these systems: detecting and interpreting the surroundings of the vehicle and the decision to take action. Regarding the former, it must be analyzed whether there are obstacles that may become potential obstacles in the path of the vehicle. The three technologies that are commonly used for long-range vehicle surroundings detection are computer vision (e.g., [5,6]), radar (e.g., [7,8]) and laser scanner (e.g., [9–12]). Sensor fusion is used to enhance the possibilities of understanding and representing the environment as well as for mitigating the deficiencies of each sensor, and several algorithms have been proposed in the past (e.g., [13–18]). There is a large set of systems and research projects that uses the information supplied by these sensors and integrate it using obstacle detection and tracking algorithms. This way in [19,20] a stereovision system that detects pedestrians has been presented, including the necessary algorithms to detect and track a pedestrian, considering whether he is in the trajectory of the car and acting on the car to stop or deviate it if necessary. Similarly in [21,22] this data fusion has been complemented with GPS-based autonomous driving. On the other hand, in [23] a complete system for detecting and tracking to avoid collisions has been presented but, only in simulation. Moreover, the popularity of digital maps means they can be used as an additional sensor [24–29], positioning the obstacles in the digital map and interacting with GPS navigation (for example, in the EU-funded project Safespot). The second aspect is how the collision avoidance system warns the driver or acts, since in the case of systems that warn the driver, this is critical to the design of the interface [30,31]. A more advanced solution considers action on the brake pedal and the steering wheel, which lies within the field of autonomous vehicles. The application of artificial intelligence techniques for the automatic management of the actuators of the vehicle enables driver assistance systems and autonomous driving systems to perform management in a similar way to human drivers while improving safety and comfort [32]. Some obstacle identification-based collision avoidance applications which fall within the pre-collision systems group are the ones presented in [20,33] to avoid accidents with pedestrians, and the theoretical proposal in [34] for overtaking slow-moving vehicles.

On the other hand, it is clear that the information retrieved by a vehicle provided only by its local sensors should be enough to prevent near accidents or to reduce the effects of a certain accident. However, new vehicle applications require additional information on the moving traffic environment from the other vehicles as well as from the infrastructure. This means that an external source of information is necessary in the vehicle itself in order to provide the necessary information to guarantee a proper performance of these assistance systems [35]. Vehicle-to-Vehicle (V2V) and Vehicle-to-Infrastructure (V2I) communications cover the gap of providing information to the Advanced Driver Assistance Systems (ADAS) from the surroundings of a vehicle [22]. Thus, a distinction is made between two generations of systems: autonomous systems and cooperative systems (vehicle-to-vehicle communications) [36]. The former are based on sensors and intravehicle communications. Cooperative systems are ones that are based on communications and an information exchange between vehicles or between vehicles and the infrastructure. This information enables the horizon that can be “seen” to be broadened so that actions can be taken [37]. The main qualitative leap between the autonomous vehicle and the connected vehicle in a cooperative environment lies in the fact that, apart from its possessing its own data and perceiving its surroundings through onboard sensors, it can receive information from other vehicles, from the infrastructure or traffic control centres. In addition, the said vehicle could, at the same time, be a source of information that could be transmitted to the outside. In this way, two-way communications are established. The potential for communications in the field of transport has become firmly established with the setting up of ambitious research programmes throughout the world, such as the European eSafety initiative, European projects such as CVIS, SAFESPOT and COOPERS, the American programmes derived from the Intelligent Vehicle Initiative and the Japanese InternetITS and Advanced Highway Systems (AHS) programmes [38].

The ongoing research and development work usually falls within one of the two groups. However, there are very few applications that encompass both aspects. The work presented in this paper is motivated by the fact that if we combine vehicles equipped with sensors and non-instrumented ones, we can generate cooperative perception systems that may extend the range of applicability of the standalone ADAS. We present an autonomous collision avoidance system, extended to other vehicles through vehicular wireless communications in order to perform cooperative perception. This ADAS perceives the environment of a leading automated car using a laser scanner and transmits the perception information to a tailing car using a Vehicular Mesh Wireless Sensor Network (WSN), whose communication technology has been specifically designed to behave as a Mesh Network, following IEEE 802.15.4 standard. This system has been designed, implemented and tested using real vehicles in the facilities of the University Institute for Automobile Research (INSIA) of the Technical University of Madrid.

## 2. Collision Avoidance System

### 2.1. General Layout

The assistance system is based on four main modules:

- Obstacle detection module: The mission of this module is to detect and track obstacles and determine their speed and direction while distinguishing between obstacles that are within the area of interest and may present a risk, and those that are outside. Positioning of the vehicle and the obstacles in accurate and detailed digital maps provide extra information.
- Decision module. This module decides the best possible action to take to avoid an accident or reduce its consequences based on the information from the surroundings. Its premise is not to generate any additional risks for other road users. This decision module should take into account the road characteristics, the own vehicle movement, the obstacles and should generate manoeuvres that are feasible in practice according to vehicle dynamics and should not be surprising for the drivers.
- Autonomous manoeuvring module. The mission of this module is to carry out the manoeuvre selected by the previous module by acting on the vehicle’s controls, without eliminating the capability of the driver of acting over those controls if the wants.
- Communications module. This module sends messages to nearby vehicles in the event of risk situations being detected that lead to avoidance measures so that those vehicles can be made aware of these anomalous moving traffic circumstances. Communications are not used for obstacles detection but to warn of sudden manoeuvres that could be performed by the autonomous system.

### 2.2. Obstacle Detection Module

The obstacle detection and tracking process comprises several phases, and improvements have been introduced in respect of previously implemented systems to increase detection reliability and avoid the system giving false negatives or positives. Described below are the phases executed by this algorithm.

#### Phase 1: Obstacle Detection and Tracking

A laser-scanner-based surroundings perception system is used to detect obstacles. It enables obstacles that the system considers may pose a risk to be detected. Specifically, a Sick LRS 1,000 long-range laser scanner is used, which, under normal working conditions can detect obstacles over 150 m distant. It uses a maximum update frequency of 10 Hz, a light aperture of up to 180° and an angular increase between beams that can reach 0.125°.

The obstacle detection algorithm comprises two fundamental blocks: an obstacle search block (grouping into sets the points comprising the obstacle detected by the laser scanner) and the obstacle tracking block. There is an extensive bibliography on this kind of algorithm (e.g., [39–42]) and by taking this as a starting point the best proposals have been adopted, the main ones being [43]:

- An iterative segmentation algorithm to avoid false groupings or the division of obstacles in complex environmental scenarios using different acceptance and rejection criteria.
- Accurate kinematic variables calculation of obstacle displacement by identifying the lateral and front axes of the obstacle.

The iterative algorithm aims to resolve the constraints found in the method proposed in [44] for segmenting the points and determining which of those points belongs to each obstacle. On the other hand, the determination requires the kinematic variables of the obstacles. This is a requirement imposed by [4] so that the pre-collision systems will work properly. This objective is achieved by implementing an algorithm to determine the main axes of the obstacle detected to obtain greater independence of the deviations in measurement and the tolerances included in the calculations.

The proposed method in [43] attempts to solve the limitations of the other methods and ensures the orthogonality of the axes of the detected vehicles at any instant. This method assumes that, in general, at least three points of the axes of a rectangular shaped obstacle that has been detected are considered to be given by two perpendicular straight lines defined by the following parameters:

- Line r_1_: Gradient: m; y-intercept: b
- Line r_2_: Gradient: −1/m; y-intercept: c

A quadratic error minimisation process is performed (giving equal weighting to the error of each side) in order to find the axes, varying the number of points belonging to each of the straight lines r_1_ and r_2_ (*n*_1_ and *n*_2_, respectively, and with *n*_1_ + *n*_2_ = *N*). The method that minimizes the quadratic error leads to the following expressions from which the gradient of the straight line r1 can be deduced:

(1) $$\begin{array}{l} m^{4}\left[n_{2}\sum_{i=1}^{n_{1}}\left(-x_{i}^{2}+\frac{1}{n_{1}}\sum_{j=1}^{n_{1}}x_{i}x_{j}\right)\right]+m^{3}\left[n_{2}\sum_{i=1}^{n_{1}}\left(x_{i}y_{i}-\frac{1}{n_{1}}x_{i}\sum_{j=1}^{n_{1}}y_{j}\right)\right]+\\ +m\left[n_{1}\sum_{i=1}^{n_{2}}\left(x_{i}y_{i}-\frac{1}{n_{2}}x_{i}\sum_{j=1}^{n_{2}}y_{j}\right)\right]+n_{1}\sum_{i=1}^{n_{2}}\left(x_{i}^{2}-\frac{1}{n_{2}}\sum_{j=1}^{n_{2}}x_{i}x_{j}\right)=0\;\;\;\text{if}\;n_{1}\neq 0\;\text{and}\;n_{2}\neq 0 \end{array}$$

(2) $$\frac{1}{m^{2}}\sum_{i=1}^{n_{2}}\left\lfloor \left(y_{i}+\frac{1}{m}x_{i}-\frac{1}{n_{2}}\sum_{j=1}^{n_{2}}\left(y_{j}+\frac{1}{m}x_{j}\right)\right)x_{i}\right\rfloor =0\;\;\;\;\;\;\;\text{if}\;n_{1}=0\;\text{or}\;n_{2}=0$$

For any possible values of m, we find b and c using the expressions in the case of *n*_1_ ≠ 0 y *n*_2_ ≠ 0:

(3) $$b=\frac{1}{n_{1}}\sum_{i=1}^{n_{1}}\left(y_{i}-mx_{i}\right)$$

(4) $$c=\frac{1}{n_{2}}\sum_{i=1}^{n_{2}}\left(y_{i}+\frac{1}{m}x_{i}\right)$$

#### Phase 2: Positioning the Vehicle

The vehicle equipped with the perception system must be positioned on the road with the purpose of determining the area of interest in which the presence of obstacles will be analyzed [43], together with the obstacle-free road areas to which the vehicle can move without danger [45]. This positioning is performed using a GPS receiver. However, numerous publications have shown that on many occasions GPS positioning is lacking in the reliability and precision required by safety applications [46], showing errors that are unacceptable for positioning on lanes or crossings. For this reason, we have used the information supplied by an RTK DGPS Topcon GB-300 receiver with an update frequency of 10 Hz and the possibility of using American GPS and Russian GLONASS that generates positions with an accuracy of less than 1 m. The GPS receiver transmits latitude/longitude positions to the computer. However, to deal with this data in an effective way it is necessary to transform it into Cartesian coordinates. In this case, Gauss-Kruger transformation is applied to the latitude/longitude coordinates to transform them into Universal Transverse Mercator (UTM) North/East Cartesian ones. The vehicle is positioned on a digital map using map-matching algorithms that ensure robust solutions even under adverse environmental circumstances [47,48].

#### Phase 3: Positioning Obstacles on the Digital Map

Finally, the obstacles must be located on the digital map so that the system can analyze which pose a risk as well as evaluating their movement over time. To do this, the angle/distance information provided by the laser scanner is transformed into UTM positions to be coherent with the GPS ones.

**Figure 1.**
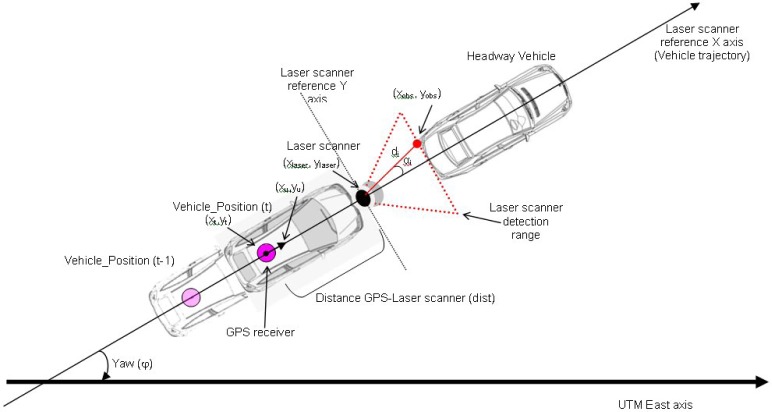
Diagram for positioning obstacles on the digital map.

To achieve this objective, two consecutive positions of this position provided by the vehicle’s orientation are taken to calculate the laser scanner position since the distance between it and the GPS receiver in the vehicle is known. This is shown in Figure 1:

(5) $$\vec{u}=\left(\begin{array}{c} x_{u}\\ y_{u} \end{array}\right)=\frac{{\vec{r}}_{t}-{\vec{r}}_{t-1}}{\left|{\vec{r}}_{t}-{\vec{r}}_{t-1}\right|}$$

(6) $${\vec{r}}_{\mathrm{laser}}=\left(\begin{array}{c} x_{\mathrm{laser}}\\ y_{\mathrm{laser}} \end{array}\right)={\vec{r}}_{t}+d\cdot \vec{u}$$

where *u⃗* represents the unitary vector in the direction of the vehicle, *r⃗* is the position of the GPS receiver of the vehicle, d is the distance between the GPS receiver and the laser scanner and *r⃗_laser_* is the position of the laser scanner. It should be pointed out that it would be possible to substitute the information from two consecutive positions of the vehicle to obtain its orientation if a gyroscopic platform is available to provide these data directly.

Once the laser scanner UTM coordinates have been calculated, we can use them to calculate the positions of the laser detected points that use the scanner as centre of coordinates, transforming angle (*α*_i_)-distance (*d*_i_) coordinates into Cartesian coordinates. In consequence, the UTM laser coordinates for the ith beam *r⃗_obs_* could be calculated as follows:

(7) $$\varphi =a\text{tan}\left(\frac{y_{u}}{x_{u}}\right)$$

(8) $${\vec{r}}_{\mathrm{obs}}=\left(\begin{array}{c} x_{\mathrm{obs}}\\ y_{\mathrm{obs}} \end{array}\right)={\vec{r}}_{\mathrm{laser}}+d_{i}\cdot \left(\begin{array}{c} \text{cos}\left(\alpha_{i}+\varphi \right)\\ \text{sin}\left(\alpha_{i}+\varphi \right) \end{array}\right)$$

Positioning of vehicle and obstacles is done on accurate and precise digital maps, developed using datalog vehicles [37,49]. The use of these kinds of maps provides the possibility of assessing free areas without obstacles where a movement is possible. It should be noted that current navigation digital maps are not accurate enough and few details for safety applications are included.

### 2.3. Decision Module

The proposed system focuses on avoiding obstacles on a single carriageway road. Therefore, the situation considered is one where a vehicle equipped with the system detects an obstacle in the lane along which it is moving. If an accident is to be avoided in this situation two manoeuvres can be performed: braking the vehicle to adapt its speed to that of the obstacle or turning the steering wheel so that the obstacle can be overtaken. Although the first option is the simplest, in circumstances where there is no other vehicle circulating in the opposite direction, a better option is to choose the second option so as not to interrupt the traffic flow. According to the vehicle surroundings information at every instant, the decision algorithm must choose the most advisable action. Vehicle surroundings include other obstacles detection or road characteristics included in the digital map, such as lane and road marking, visibility distance, *etc.* Deterministic vehicle tracking models are used for this purpose [50].

The algorithm’s premise is to calculate the minimum distance that will ensure a safe action. Firstly, the minimum distance at which the deceleration action must be begun to adapt the speed to that of the obstacle *v_obs_* is calculated. If a constant deceleration of *a* is assumed, the distance required for this reduction in speed is given by the following expression:

(9) $$d_{\mathrm{braking}}=\frac{v^{2}-v_{\mathrm{obs}}^{2}}{2a}$$

**Figure 2.**
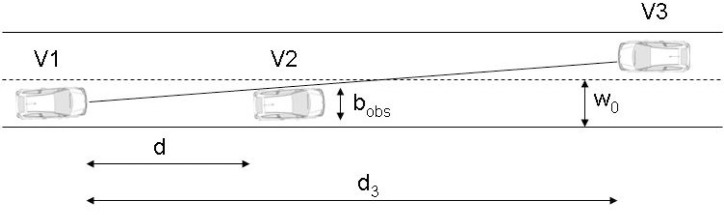
Scenario for the decision whether or not to overtake the slow vehicle.

The overtaking manoeuvre is also analyzed. The algorithm calculates whether the manoeuvre can be completed before the path of another vehicle moving in the opposite direction along the left-hand lane is interfered with. The manoeuvre comprises three phases: a lane change, moving along the parallel lane and returning to the initial lane. A scenario has been put forward where vehicle V1 with the laser scanner is moving at a constant speed along a straight lane where it encounters vehicle V2 at a distance d moving at a lower speed, while another vehicle V3 may be moving in the opposite direction along the left-hand lane at a distance d_3_ (Figure 2).

So the overtaking manoeuvre can be carried out, the sum of the distances travelled by vehicle 1 and vehicle 3 during the total time of the manoeuvre *t_T_* must be less than or equal to the distance d_3_, so that both vehicles complete their manoeuvres in the same cross section of the road:

(10) $$\left(v_{1}+v_{3}\right)\cdot t_{T}\leq d_{3}$$

where *v_i_* is the speed of vehicle *i*.

On the other hand, the distance travelled by vehicle V1 until it overtakes vehicle V2 *d_s_* and the time taken *t_s_* are given by:

(11) $$d_{s}=d+v_{2}\cdot t_{s}+L_{1}+L_{2}=v_{1}\cdot t_{s}$$

(12) $$t_{s}=\frac{d+L_{1}+L_{2}}{v_{1}-v_{2}}$$

where *L_i_* is the length of vehicle *i*. In addition, *t_s_* includes the lane-change time *t_LC_* and the time taken by vehicle V1 moving along the left-hand lane, while *t_T_* includes the previous time *t_s_* and the second lane-change, that is:

(13) $$t_{T}=t_{s}+t_{LC}$$

Therefore, by taking Equations (12) and (13) in Equation (10), an upper limit for the speed of V2 is obtained:

(14) $$v_{2}\leq v_{1}-\left(v_{1}+v_{3}\right)\cdot \frac{d+L_{1}+L_{2}}{d_{3}-\left(v_{1}+v_{3}\right)\cdot t_{LC}}$$

Also to be taken into account is the fact that the distance d between V1 and V2 must be greater than a value that will allow the lane change to be made safely. This fact is reflected in the following equation:

(15) $$d\geq t_{LC}\cdot \left(v_{1}-v_{2}\right)$$

From which the minimum speed for V2 can be deduced:

(16) $$v_{2}\geq v_{1}-\frac{d}{t_{LC}}$$

If the system detects that the speed v_2_ measured by the laser scanner confirms the disparities in Equations (14) and (16), it will decide to overtake. It should be noted that all the variables are known, which means that at every instant the algorithm assesses whether or not it is possible to overtake.

However, the fact that no obstacle is detected in the left-hand lane does not necessarily mean the overtaking manoeuvre can be carried out. For instance, if no obstacle is detected in the left-hand lane in the laser’s field of vision, in the most unfavourable extreme case, V3 could be found to be in the initial instant at a distance *d_laser_* from V1. That most unfavourable distance is the minimum between the scanner’s maximum range and the field of vision allowed by the obstacles. Geometrically it is possible to establish what the field of vision is by taking the fact that vehicle V2 is an obstacle that impedes part of the vision. For safety reasons, a vehicle is deemed to be recognized when data are received from at least half its front part. The laser’s visibility distance is then defined by the following expression:

(17) $$d_{\mathrm{laser}}=\frac{2\cdot w_{0}}{b_{\mathrm{obs}}}\cdot d$$

where *b_obs_* is the obstacle width and d is the distance between the scanner and the obstacle. In this situation, in equation (A), *d*_3_ should be substituted for *d_laser_* and a hypothesis must be made regarding the speed of V3, which, reasonably, should be related to the road’s speed limit.

Finally, the algorithm must estimate the lane-change manoeuvre. Different models exist for calculating the lane-change manoeuvre. For example, in [51] for carrying out a lane-change manoeuvre is calculated by taking into account the maximum lateral acceleration and over-accelerations defined in [52]. The distance travelled by the vehicle during that lane change is the product of the manoeuvre time multiplied by the speed, taken as constant, which gives the following expression:

(18) $$D_{LC\_\text{min}1}=v\cdot T_{LC}=v\cdot \left(\frac{a_{\text{max}}}{J_{\text{max}}}+\sqrt{{\left(\frac{a_{\text{max}}}{J_{\text{max}}}\right)}^{2}+4\cdot \frac{w_{0}}{a_{\text{max}}}}\right)$$

where *D_LC-min1_* is the minimum lane-change distance; v the longitudinal speed; *T_LC_* is the minimum lane-change time; *a_max_* is the maximum lateral acceleration; *J_max_* is the maximum lateral over-acceleration and *w*_0_ is the maximum lateral displacement during the manoeuvre.

Other works along the same lines are included in [53], where the lane-change manoeuvre approximates to a sinusoidal path:

(19) $$y=A_{1}+A_{2}\cdot \text{cos}\left(A_{3}\cdot x\right)$$

In this case, the lane-change distance is given by the expression:

(20) $$D_{LC\_\text{min}2}=\pi \cdot v\cdot \sqrt{\frac{w_{0}}{2a_{\text{max}}}}$$

In the same way, the same authors propose the lane-change distance if this manoeuvre is modeled according to an exponential approximation, the result being:

(21) $$y=A_{1}+\frac{A_{2}}{1+\text{exp}\left(A_{3}\cdot \left(x-A_{4}\right)\right)}$$

(22) $$D_{LC\_\text{min}3}=v\cdot \sqrt{\frac{4.142\cdot w_{0}}{a_{\text{max}}}}$$

Other works along the lines of defining controllers for developing lane change or overtaking manoeuvres can be found in [54–58], among others.

The above figures have been compared and validated by on-track tests because of the importance of that distance when computing the total distance required for the overtaking manoeuvre. These tests involved 4 drivers who carried out lane change manoeuvres at different speeds, measuring the distance travelled from the start of the steering wheel manoeuvre until reaching the parallel lane. To this end, a vehicle was instrumented with a Trimble R4 RTK GPS receiver, a L-CE Correvit non-contact speed sensor, an RMS FES 33 Gyroscopic platform (that provides accelerations and yaw angle) and a Bus Can Vector CANcaseXL interface (that provides steering wheel angle). The results for the 50 km/h case study (with a 2% maximum variation) are set out in Table 1. A good correlation can be seen between the models and experimental data although with a slight underestimation of the distance by the models. This fact is taken into account by the decision module in order to adopt larger safety margins and theoretical and experimental results are combined.

### 2.4. Autonomous Manoeuvring Module

After the risk assessment, the system decides whether or not some action is required to avoid a collision by comparing the different possible options. Then, the automated vehicle controls let the manoeuvre be performed should the driver not react in the right way. In this sense, within the collision avoidance system, the obstacle detection algorithm acts as a high-level layer that generates orders to the low-level layer that acts on the vehicle’s controls if necessary.

Most of the collision avoidance systems developed use brakes or speed reduction as the main action [59,60]. However, in many situations this action is not enough or is not the most appropriate one, and the control system has to additionally take control of the steering wheel in order to avoid the accident. The developed vehicle control architecture has been implemented and installed in a Citroën C3 Pluriel testbed vehicle that equips an automatic gearbox, whose actuators (accelerator, brake and steering wheel) have been automated and prepared to be controlled from the onboard collision avoidance system.

The vehicle equips an electronically actuated throttle. The engine central unit controls the fuel inlet by considering the voltage signal that it receives depending on the accelerator position. The solution used is to bypass the electrical signal given from the pedal by one generated from an Advantech USB-4711A acquisition card. To switch between the manual action and autonomous one, a switching relay is used. The vehicle brake has no electric power assistance and the solution that has been implemented for automating this system is direct action on the brake pedal via an external actuator. The braking module consists of a set of a Maxon RE35 DC motor and an ENC HEDL 5540 encoder, controlled by the Maxon EPOS 24/5 position controller that receives the target from the low-level control system. The motor acts on a pulley that moves the pedal. The speed control loop is closed by the speed measurement from the vehicle CAN bus.

On the other hand, the steering system is electrically assisted. This system consists of an electric motor attached to the steering rack through a gear. This motor exerts a power over the steering that is proportional to the power exerted by the driver over the steering wheel, measured through a torque sensor located in the steering bar. This signal is received by a control/power controller that sends a PWM signal to assist the steering movement so that very little effort is required of the driver and this system has been used for automating the steering system of the vehicle. The control unit generates a signal which passes through the Maxon ADS 50/10 4-Q-DC Servo amplifier that is responsible for controlling the vehicle steering motor. The feedback loop control is performed through the signal provided by the wheel rotation sensor included in the steering column. Switching between manual and automatic control is performed by a power relay box.

A central unit manages the vehicle actuators in order to comply with the commands sent by the high-level controllers. This is a low-level layer that is able to receive signals from different sources. This automation architecture makes it possible to control the vehicle by considering signals provided by the collision avoidance system or other inputs for autonomous driving. Two low-level control systems have been designed in order to satisfy the mission of the actuator control: steering controller and speed controller. The first must be able to receive steering angle commands from a high-level controller and to send the coherent signals to the actuators to meet these orders. Similarly, the second controller must be able to receive the desired speed commands from a high-level system and send the necessary orders to the accelerator and brake pedals to achieve this speed. It should be kept in mind that steering and speed are influenced by many internal and environmental factors that give rise to complex dynamics that are difficult to model with a classic method. The solution adopted to manage these elements is the application of fuzzy logic. Two fuzzy controllers have been designed to support the vehicle’s autonomous driving: the steering controller, whose input variables are the position error (difference between the target steering position and the real position), the steering position and the speed of the car, and the speed controller, whose fuzzy input variables are the speed error (difference between the target and the real speed) and the acceleration.

Considering the proposed architecture, the driver has control of the brake system and can decelerate the vehicle when they want but they cannot provide lower decelerations than the signal that the system proposes. When the automatic mode is activated, the driver loses control of the accelerator pedal but does not completely lose control of the steering of the vehicle, but no assistance is available for them. Furthermore, vehicle automation has been performed so that it is possible externally to stop the vehicle at any time via a remote control in the event of detecting anomalous behaviour in the tests. Finally, the design is intended to be general and serve as a low-level system that could be used by any assistance system.

### 2.5. Communications Module

Since the above action has been generated through the detection of a risk situation, the assistance system issues a warning to alert any vehicles circulating near it. Hence, once the emergency manoeuvre is selected by the automated vehicle ADAS, a warning is set to those other vehicles that are circulating in the driving area. This signal includes the GPS position, identifier and speed of the signalling vehicle and a timestamp to validate the message confidence. Every vehicle is also synchronized with the others since they equip a GPS receiver and use the GPS time to perform the necessary latency and validity calculus. With this information, the vehicle that receives the emergency signal will show a message in a human machine interface, so the driver is alerted in advance of the risk situation and would have more time to reduce speed if necessary.

The communications system is based on wireless mesh networks, whose fundamental feature is that their topology can be automatically reconfigured at every instant, while always supporting an available route for the transmission of the information among network nodes in such a way that they form a VANET (Vehicle *Ad-hoc* Network).

The Vehicle-to-Vehicle communication mesh devices are Maxfor Inc. MTM-CM3100 gateways based on the TelosB platform. They are used as interface to access the vehicular mesh network. This device works under the TinyOS open code operating system. In order to access the wireless network at 2.4 GHz it uses the IEEE 802.15.4 standard at physical and link level and a mesh routing protocol, which guarantees the desired functionality of the VANET. The protocol implemented in TinyOS for the mesh routing reconfigures the network in accordance with the root mean square (RMS) value of the different network nodes signal. As shown in [61], the latency of the mesh network is less than 1 ms, in direct connection one hop and two hops. The number of lost messages in mobility is 6.25% at speeds up to 50 km/h and the mesh structure is reconfigured only when one node loses the connection with its established route, using the transmission and reception powers as parameters to take this decision.

## 3. Tests

### 3.1. Test Scenarios, Vehicles and Instrumentation

**Figure 3.**
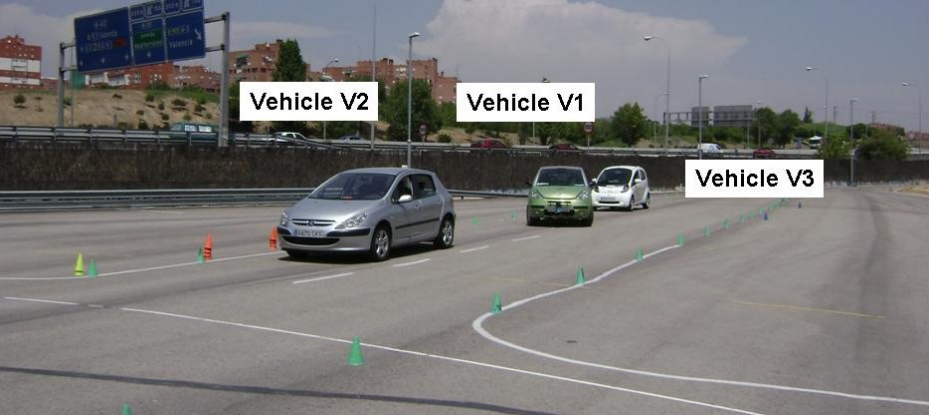
Scenario 1.

A description of test scenarios for collision avoidance systems can be found in many publications (e.g., [62–64]). The system presented in this paper focuses on detecting obstacles on a single carriageway road. The scenarios considered are as follows:

(1) Scenario 1 (Figure 3):

The vehicle equipped with the assistance system (V1) detects another vehicle V2 moving in front of it along the same lane at an abnormally low speed (or it is stopped). Another vehicle, V3, is moving behind V1. As the left-hand lane is free, the avoidance collision action will be to turn the steering wheel to let V1 overtake V2. In turn, at the start of the manoeuvre, V3 will receive a warning to alert the driver so that they can adapt their speed to that of V2 with advance notice.

**Figure 4.**
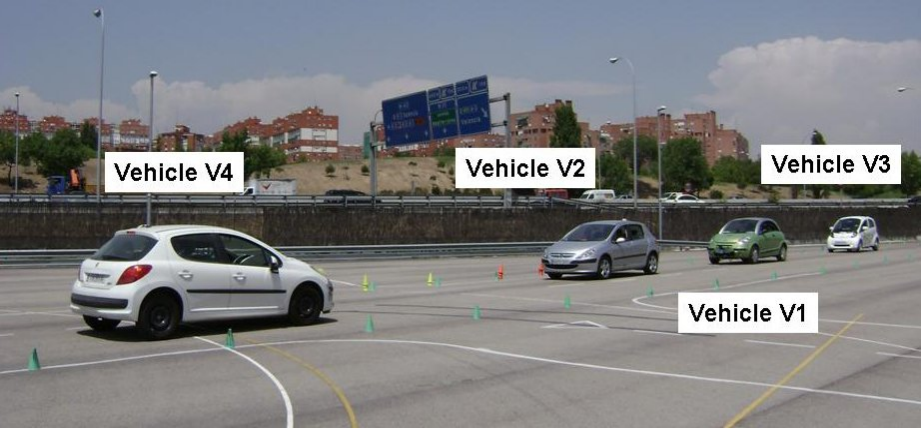
Scenario 2.

(2) Scenario 2 (Figure 4):

The second situation contemplates a similar scenario that is modified by the fact that another vehicle, V4, is approaching along the left-hand lane and is detected by the perception system of V1. In such circumstances, it is not possible for V2 to overtake and the system’s decision must be to adapt the speed of V1 to that of V2. As in the above case, V3 will receive a warning from V1.

The tests took place at the facilities of the University Institute for Automobile Research of the Technical University of Madrid. It has a test track where a straight section of road with two lanes is marked out, which is where the manoeuvres are carried out.

The scenarios contemplated involve three (in the first case) or four vehicles (in the second). The instrumentation and signals measured in each of the vehicles to analyze the manoeuvres are set out in Table 2. It should be noted that part of the instrumentation included is not necessary for the system to function, the only essential part being that included in vehicle V1.

### 3.2. System Performance

**Figure 5.**
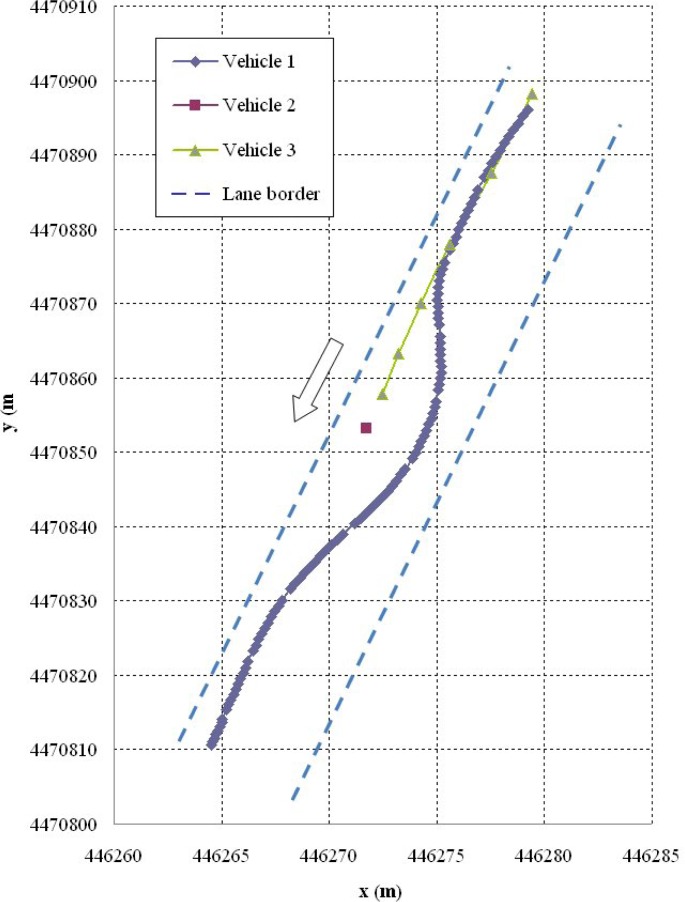
Vehicle paths during the manoeuvre.

**Figure 6.**
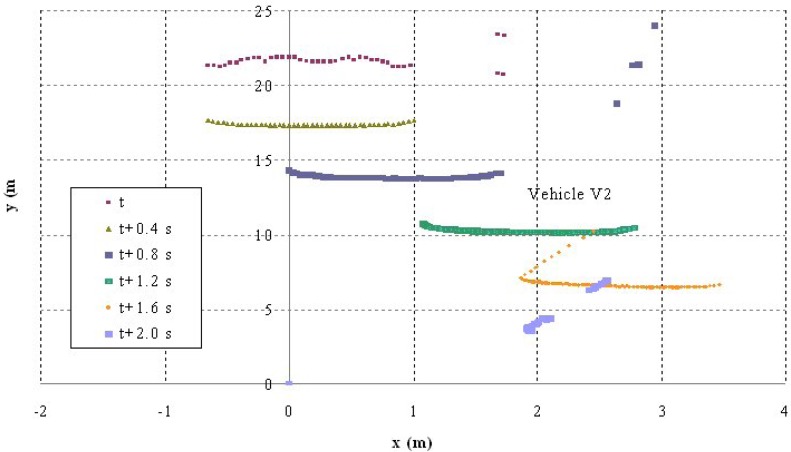
Laser scanner detection during the test.

**Figure 7.**
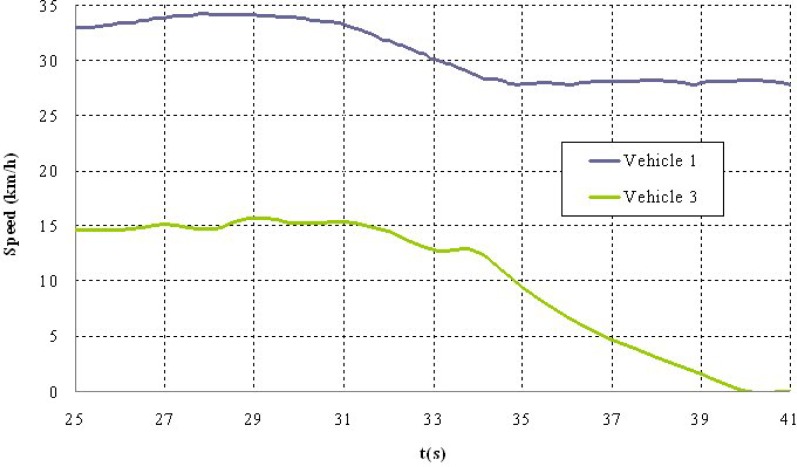
Vehicle speeds during the test.

**Figure 8.**
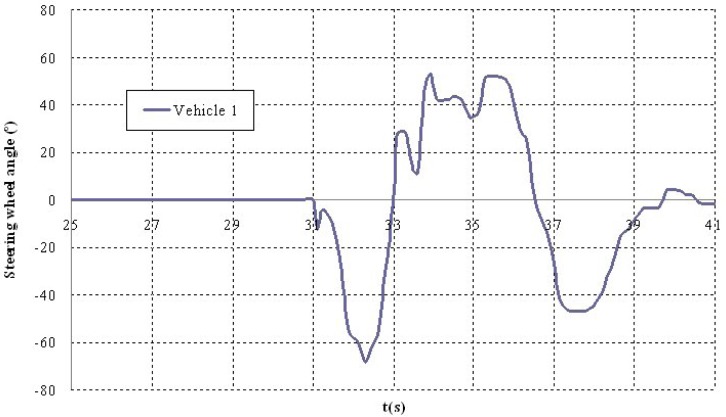
Steering wheel turning angle during the test.

Shown below are the results of two of the tests carried out. The first corresponds to a scenario 1 manoeuvre (Figure 5) in which vehicle V2 is stopped in the lane. Vehicle V1 is approaching it at a speed of over 30 km/h and automatically overtakes as no other obstacles have been detected in the left-hand lane to impede it (Figure 6). It should be noted that the position relative to the scanner of the points detected for obstacle V2 varies in the two coordinates. This is because as vehicle V1 approaches, it also overtakes leaving the obstacle to its right. However, at the instant vehicle V1 starts its manoeuvre it issues a warning to vehicle V3 moving behind it, whose driver proceeds to reduce speed until it stops behind vehicle V2 (Figure 7). Finally, Figure 8 shows how the steering wheel of vehicle V1 evolves during the automatic overtaking manoeuvre.

**Figure 9.**
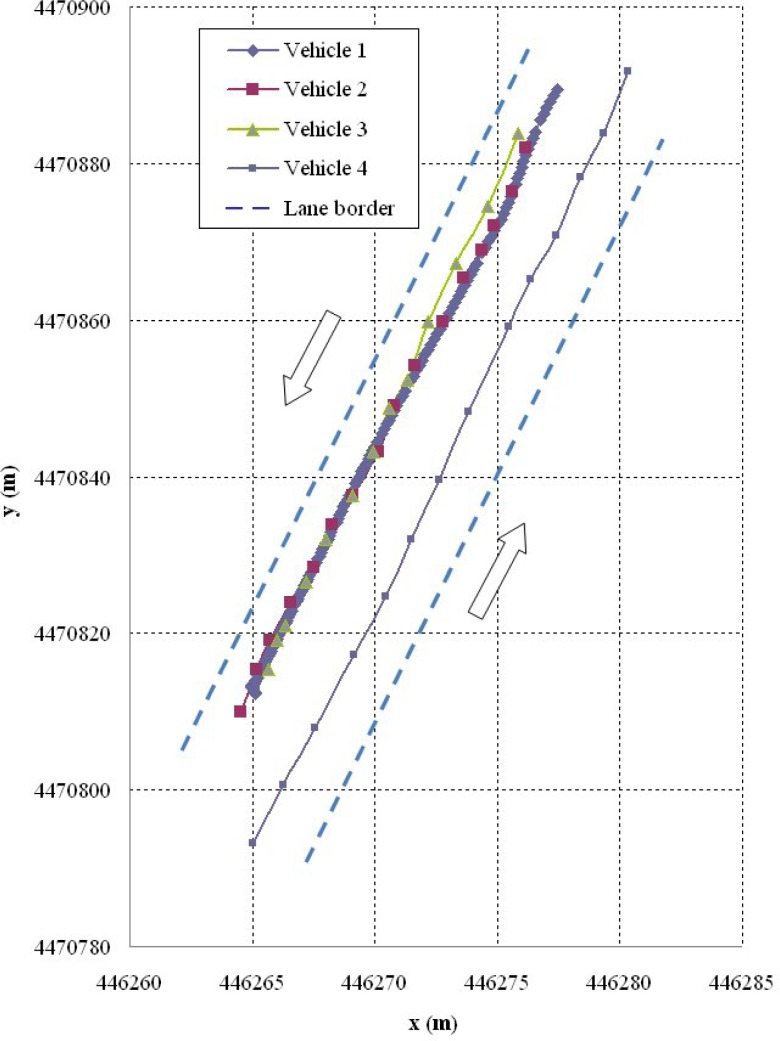
Vehicle paths during the manoeuvre.

**Figure 10.**
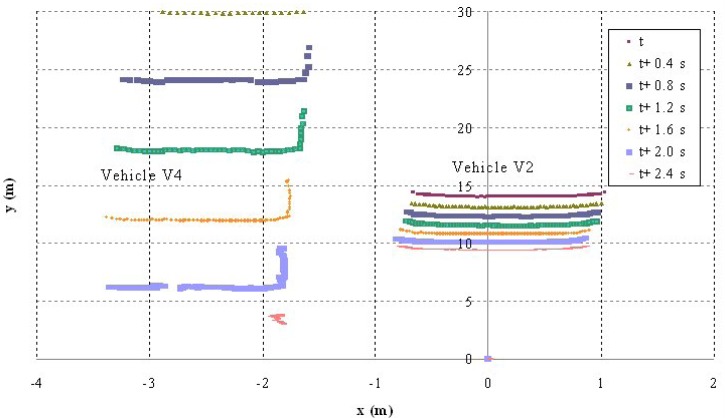
Laser scanner detection during the test.

**Figure 11.**
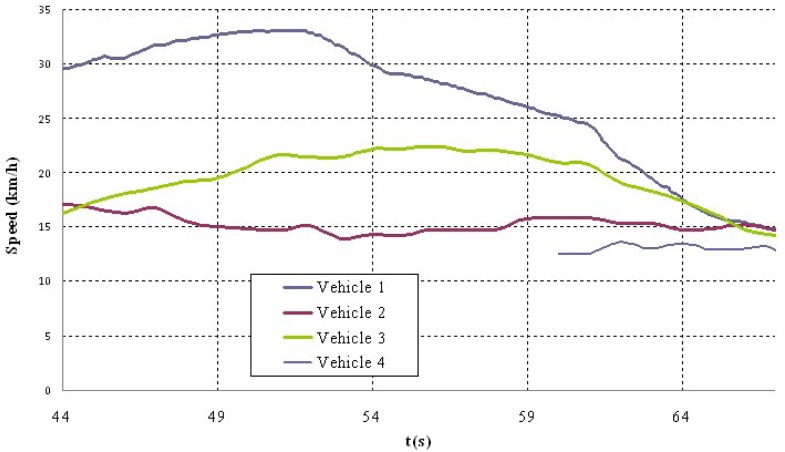
Vehicle speeds during the test.

**Figure 12.**
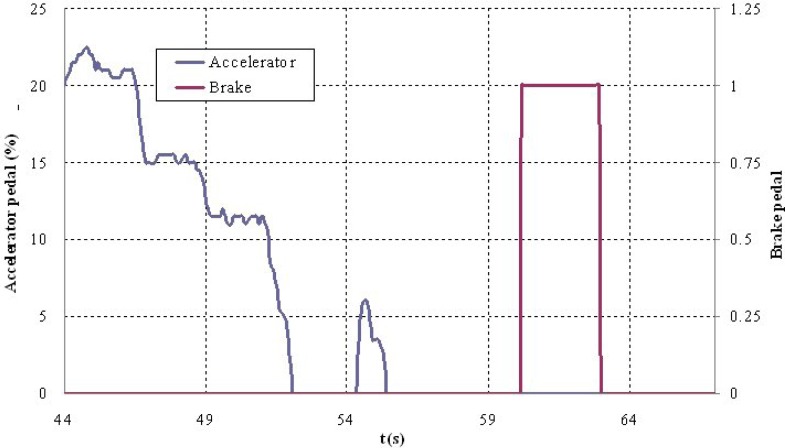
Steering wheel turning angle during the test.

The second test corresponds to scenario 2 in which vehicle V2 is moving at an abnormally slow speed and vehicles V1 and V3 are approaching from behind (Figure 9). Vehicle V1 detects that obstacle but also identifies another vehicle V4 moving along the left-hand lane in the opposite direction (Figure 10), which prevents the vehicle from overtaking. For that reason, it automatically reduces speed to adapt its speed to that of vehicle V2 and issues a warning to vehicle V3 that is following it and whose driver also proceeds to adapt their speed (Figure 11). The automatic action on the brake pedal of vehicle V1 (Figure 12) is consistent with the previous speed reduction.

Finally, it should be noticed that in the case of the experiments related to the present paper, three vehicles are connected to the wireless network, and they circulate consecutively at distances of less than 100 m at speeds up to 50 km/h. In consequence, the real time performance of the network is warranted. The above examples make it clear that the assistance system works properly on three levels:

- Obstacle detection and monitoring, identifying obstacles that pose a risk.
- Decision as to the most appropriate manoeuvre to be carried out in each scenario depending on how the surroundings are interpreted.
- Execution of the collision warning manoeuvre.

## 4. Conclusions

In this paper we have presented a driver assistance system that involves pre-collision actions with obstacle detection and vehicle-to-vehicle communication to alert the driver of danger situations. To this end, the controls of a vehicle were automated. The vehicle was also equipped with a laser scanner technology-based vehicle surroundings perception system and with a wireless communications module.

The practical application was focused on a system that activates in the face of obstacles in the lane on a single-carriageway road, where there are two possible actions: braking or avoidance. Combining a pre-collision avoidance system with a cooperative warning represents an innovation over other already developed systems. For example, pre-collision systems can give rise to sudden manoeuvres that pose a risk to other road users. However, in the developed system the whole vehicle environment is assessed so that the best possible action can be chosen and other users warned of the risk. On the other hand, if we are dealing with systems that detect vehicles or obstacles in general, give rear-end collision warnings or warn of vehicles that are moving abnormally slowly or are stopped on the road, if the system is autonomous it only detects the obstacle but does not alert other road users who may be caught unaware of the same obstacle. However, if the detection is done by communications between vehicles all of them need to be equipped with communication systems. A similar situation arises in assistance systems for overtaking. If these are communication-based then the system needs to be widely implemented in the vehicle stock. This is not the case with the proposed system since obstacle detection is based on autonomous sensors and communications are only used to alert other drivers of the fact that the vehicle with the collision avoidance system is going to perform an evasive manoeuvre to avoid an obstacle.

Finally, a system such as the one developed enables early warning alerts to be given to other road as warnings can be given after detecting the risk but before carrying out the avoidance manoeuvre. Hence, this is a basic ADAS but a good example to demonstrate the benefits of combining a standalone headway vehicle detection system with automatic actuation with a communication system to transmit an emergency signal, generating a cooperative perception system. This combination tries to take advantage of each system, considering the fact that the system should be useful for the vehicle that includes it from the beginning, independently of the market penetration, and would help others users in case they include the appropriate receivers.

The proposed system applies the obstacles detection and tracking algorithms that have been previously proved and better results than previous techniques have been obtained [43]. Furthermore, apart from the detection of obstacles and their positioning on a digital map according to the knowledge of the accurate position of the vehicle with the system, accurate and detailed digital maps [37,49] are used for assessment of free areas where movement of the vehicle is safe enough. This fact improves de decisions module capabilities in relation to some previous works because information increases and the possibility of wrong maneuvering decisions diminish. The decision module for assessing overtaking maneuvers is based on theoretical models, but experimental tests have been carried out in order compare results in lane change maneuvers and adopt decisions that could be actually implemented in a real situation. Finally, the communications module is used to alert other drivers, because some automatic actins could be severe and intrinsically imply some sink to vehicles that are moving behind the one with the system. This is a module that works independently but improves the functionality of the whole system. Obviously, communications between vehicles allow earlier detection of hazardous situations, but above cited limitations reduce their effectiveness.

There are two main future lines of work which are currently being worked on. On the one hand, the environment perception system needs to be enhanced with other short and long range sensors to furnish a more reliable, complete and robust representation of the vehicle surroundings. By so doing, obstacle detection would not be confined to the vehicle’s headway zone but the presence of obstacles would need to be assessed near to the sides or rear of the vehicle. Incorporating more sensors means using the sensorial fusion algorithms mentioned previously.

On the other hand, we are looking to generalize the system’s decision module so it will be able to come up with collision avoidance manoeuvres in more general and complex scenarios. This evolution of the system is linked to the use of artificial intelligence techniques so that the system will be capable of analyzing situations in a similar way to a human driver and make decisions as a driver would, but avoiding errors of perception, decision or action [65].

## Figures and Tables

**Table 1.** Distance of the lane change manoeuvre at 50 km/h.

**Results**	**Average value (m)**	**Standard deviation (m)**	**Difference between model and experimental data**
*D*_*LC_min* 1_	22.95	-	−11.12%
*D*_*LC_min* 2_	23.90	-	−7.44%
*D*_*LC_min* 3_	21.90	-	−15.18%
Experimental data	25.82	5.33	-

**Table 2.** Vehicle instrumentation.

**Vehicle**	**Instrumentation**	**Information**
V1: Vehicle including the surroundings detection system	Laser scanner Sick LRS 1000	Obstacle position
	Trimble R4 RTK GPS receiver	Position on the digital map
	RMS FES 33 Gyroscopic platform	Longitudinal and lateral accelerations, yaw angle
	Bus Can Vector CANcaseXL interface	Speed, steering wheel, accelerator pedal and brake pedal
	Wireless communication module	Warnings issued to nearby vehicles
	Human Machine Interface	
V2: Vehicle detected as obstacle	Topcon GB-300 GPS receiver	Position
	Bus Can Vector CANcaseXL interface	Speed
V3: vehicle moving behind the system-equipped vehicle	Garmin GPS eTrex H receiver	Position
	Bus Can Vector CANcaseXL interface	Speed
	Wireless communication module	Receipt of warnings issued by V1
	Human Machine Interface	
V4: vehicle moving along the left-hand lane in the opposite direction	Astech G-12 GPS receiver	Position
	Wireless communication module	Receipt of warnings issued by V1
	Human Machine Interface	
